# Application of Quality by Design Principles to the Development of Oral Lyophilizates Containing Olanzapine

**DOI:** 10.3390/pharmaceutics15071967

**Published:** 2023-07-17

**Authors:** Maja Bjelošević Žiberna, Pegi Ahlin Grabnar

**Affiliations:** Faculty of Pharmacy, University of Ljubljana, Aškerčeva c. 7, 1000 Ljubljana, Slovenia; maja.bjelosevic.ziberna@ffa.uni-lj.si

**Keywords:** oral lyophilizate, lyophilization, quality by design, disintegration time, Minitab^®^

## Abstract

Oral lyophilizates are intended for application to the oral cavity or for dispersing in water. The purposes of this research were: (i) to set up the quality by design approach in the development of oral lyophilizates for drug incorporation; and (ii) to evaluate the established approach by comparing its outcomes with experimentally obtained results. Within the knowledge space, properties about drugs, excipients, and the lyophilization process were acquired, followed by the determination of critical quality attributes via risk identification. Risks were assessed by failure mode and effective analysis, which recognized critical material attributes, i.e., type, concentration, particle size, solubility of drug and excipients, while as main critical process parameters, cooling rate, shelf temperature, and chamber pressure during drying were pointed out. Additionally, design space was established using the Minitab^®^ 17 software and valued with an 88.69% coefficient of determination. A detailed comparison between the model and experimental results revealed that the proposed optimal compositions match in the total concentration of excipients (6%, *w*/*w*) in the pre-lyophilized liquid formulation, among which mannitol predominates. On the other hand, a discrepancy regarding the presence of gelatin was detected. The conclusion was that the set model represents a suitable onset toward optimization of drug-based oral lyophilizates development, preventing unnecessary investment of time and resources.

## 1. Introduction

The European Pharmacopoeia defines oral lyophilizates (OLs) as solid pharmaceutical forms intended for application to the oral cavity or for dispersion/dissolution in water before ingestion [1]. Due to their rapid disintegration and drug dissolution, the rate of absorption is improved, thus enabling the immediate onset of the therapeutic effect [2,3]. One of the advantages of OL is that fluid intake or chewing is not required, so consumption is facilitated, which leads to improved patient compliance. The advantages of OLs are especially important for chronic patients, especially geriatric and pediatric patients. Due to the beneficial properties of OLs, their use is becoming increasingly popular, even with patients who do not have major swallowing problems. However, OLs have some disadvantages, such as poor mechanical resistance due to the high hygroscopicity, high porosity, and low density of their structure.

OLs are less suitable for drug substances that are incorporated in high doses, as the optimal content is usually up to 20 mg. In addition, the total weight of the OL should not exceed 500 mg. Typically, active pharmaceutical ingredients (APIs) with poor solubility in water are incorporated into OLs. More precisely, for water-insoluble or poorly water-soluble drugs, the maximum amount of drug substance is 400 mg, and for water-soluble API, a lower dose of up to 60 mg is allowed. Water-soluble drugs can have a plasticizing effect on the matrix of OLs, which critically affects the quality of OLs and the duration of the lyophilization process. Poorly soluble or insoluble APIs allow easier incorporation into the formulation by preparing aqueous suspensions, which require the addition of appropriate excipients, e.g., gelatin. Drugs for which fast absorption is desirable are usually analgesics, antihistamines, antiepileptics, anxiolytics, and sedatives. Olanzapine is a very poorly water-soluble antipsychotic, primarily indicated for the treatment of schizophrenia. Additionally, it is effective in the treatment of bipolar disorder, especially as maintenance therapy. Considering all the above, olanzapine was selected as our model drug.

To cover the unpleasant taste of active substances (and excipients), usually sweeteners and flavor modifiers are incorporated [4,5,6,7]. OLs consist of excipients such as binders (e.g., gelatin, xanthan gum, methylcellulose, hydroxypropyl methylcellulose, dextrin, arabic gum, guar gum, agar, polysaccharides, alginate, dextran, polyvinylpyrrolidone (PVP), etc.), matrix-forming agents/fillers (e.g., mannitol, maltodextrin, isomalt, etc.), and matrix stabilizers/disintegration enhancers (e.g., sugars such as sucrose, sugar alcohols such as mannitol and sorbitol, L-amino acids, etc.). The choice of the appropriate components and their concentrations depend mainly on the desired properties of the final product. It is important that excipients are thermostable and suitable for dispersion preparation. OLs are manufactured by the lyophilization process [8].

Lyophilization is a drying method where solvent (mostly water) is removed from a frozen sample by sublimation. The lyophilization process consists of a freezing phase (and an annealing phase), primary drying, and secondary drying. Drying is performed at low temperatures and pressure. It enables the formation of a highly porous structure. The conditions of the process must be carefully planned, as they can significantly affect its duration and the properties of the final product, namely disintegration time [4,8,9]. Lyophilization is the method of choice when thermolabile active substances are being used. It is, however, a very expensive, time-consuming, and complex process [10]. The first approved pharmaceutical product on the market in the form of OL was Claritin RediTabs (Schering-Plough, Kenilworth, NJ, USA) in 1996, manufactured using Zydis^®^ lyophilization technology (Catalent Pharma Solutions, Somerset, NJ, USA) [6].

Quality by design (QbD) is a systematic approach to development that begins with pre-defined goals and emphasizes the importance of the product, manufacturing process, and process control understanding. It is based on the concept that quality cannot be ensured by final product testing but should be built into the product. The QbD approach consists of several elements, such as prior knowledge use, design of experiments (DoE), quality risk management, and knowledge management throughout the entire life cycle of a drug product, in addition to a variety of steps that are described in the International Council on Harmonization of Technical Requirements for Registration of Pharmaceuticals for Human Use (ICH; Q—Quality Guidelines) Q8, Q9, and Q10 guidelines. These include the definition of the quality target product profile (QTPP), identification of quality attributes and selection of critical quality attributes (CQA), selection of the manufacturing process method, identification of critical process parameters (CPP), critical material attributes (CMA), and quality risk assessment. QTPP is a summary of all characteristics of a pharmaceutical product to ensure its quality and, thus, its safety and effectiveness. CQAs are the physical, chemical, biological, or microbiological properties of the final product that must be within a certain range to ensure the desired QTPP. CMAs are physical, chemical, biological, and microbiological characteristics of materials used, while CPPs stand for process parameters whose variability critically affects the properties of the final product, which must be monitored and controlled to achieve the desired product quality. DoE is an important part of QbD and is based on the results of risk assessment [11]. It facilitates the establishment of the design space, i.e., the multidimensional combination and interaction of factors that have been shown to provide quality, and specifies information on the values of the factors within which the desired values of responses can be expected [12,13]. Quality risk management is a systematic process of assessing, monitoring, communicating, and reviewing pharmaceutical product quality risks throughout their entire life cycle and begins with risk assessment [11,14]. Risk assessment (the combination of the probability of an event and the severity of the damage it may cause) is based on scientific principles, and its main goal is to ensure patient safety. Usually, risk assessment is repeated when more information and knowledge are gained. Failure mode and effects analysis (FMEA) is one of the tools for methodologically assessing, understanding, and documenting potential risks to a process and product quality. It is used for the purpose of quantitative risk assessment, which allows for the identification of factors that are most likely to adversely affect the product and/or its manufacturing process [14].

The main purpose of this study was to implement the QbD perspective in the development of oral lyophilizates to avoid further trial and error, which is especially important when incorporating drugs. Oral lyophilizates are pharmaceutical dosage forms whose development is a consequence of the aging population and the increasing prevalence of psychiatric and neurodegenerative diseases. At the same time, the composition of lyophilizates directly depends on the properties of the drug substance, so considering the fact that many new drug substances are poorly soluble, oral lyophilizates represent one of the possible solutions. The development of oral lyophilizates is related to the specific drug substance and, consequently, to adjustments in the production process and the establishment of the QbD system. To the best of our knowledge, there are no studies published in the literature that describe the development of oral lyophilizate with olanzapine from a QbD point of view, combining risk assessment and design space establishment. Based on the experimentally obtained results regarding lyophilizate composition, appearance, and disintegration time, the design space, along with model set-up, was established using Minitab^®^. The optimized compositions of oral lyophilizates proposed by the model were compared with the most suitable oral lyophilizates obtained throughout the laboratory work.

## 2. Materials and Methods

### 2.1. Materials

Gelatin, polyvinylpyrrolidone (PVP) K25, and olanzapine were obtained from Sigma-Aldrich, St. Louis, MO, USA, and mannitol was purchased from Merck, Darmstadt, Germany. Ultra-pure water was obtained from a Milli-Q purification system (A10 Advantage; Millipore Corporation, Bedford, MA, USA).

### 2.2. Methods

#### 2.2.1. Sample Preparation

At the beginning, a half amount of water was weighed in a beaker, and excipients (mannitol or PVP K25) were gradually added during constant mixing. In the case of gelatin, the water was heated between 60 and 70 °C first, then gelatin was added. At the end, the rest of the water was added, and 2 mL of each sample was aliquoted in a 10 mL beaker and held at −20 °C for 24 h before lyophilization (Table 1). When preparing OLs with API, olanzapine, in final concentration of 2.5 mg/mL, was added after the total amount of water and mixed for 15 min. Before storage at −20 °C, formulations with API were frozen in liquid nitrogen (−196 °C) to avoid API sedimentation.

#### 2.2.2. Lyophilization Process

Lyophilization was conducted in a laboratory freeze-dryer (Epsilon 2-6D; Christ, Osterode am Harz, Germany) equipped with a capacitance manometer (722B Baratron; MKS Pressure & Vacuum Measurement Solutions, Andover, MA, USA). The beakers containing the formulations were placed in the middle of the shelf and surrounded by a row of placebo vials. The freezing procedure was kept constant throughout the cycles, with the shelf temperature ramped to −40 °C at a rate of −0.5 °C/min. In the case of drug incorporation, to prevent melting of the frozen samples and destabilization of the API suspension, lyophilizer was cooled down first, and then the samples were placed on the shelf. During primary drying, the shelf temperature was set to 20 °C and chamber pressure to 0.10 mbar. In secondary drying, the shelf temperature was raised to 40 °C, and chamber pressure was the same as during primary drying. The graphic display of the shelf temperature and chamber pressure was provided by Christ LPC-32 (LSC) SCADA version 2.4.0.2. software, which enables process monitoring, which is particularly important for determining the completion of primary drying step. The criteria were based on the concept that, when product temperature approached the shelf temperature, this indicated the end of the primary drying. For accurate analysis of the lyophilization process, the recorded cyclical data were used.

#### 2.2.3. Product Appearance

Oral lyophilizates were visually evaluated after each lyophilization cycle according to whether they were “acceptable” or “collapsed.”

#### 2.2.4. Disintegration Time

Disintegration time of oral lyophilizates was measured according to the European Pharmacopoeia, 10th edition. Lyophilizates were placed in 200 mL of water at a temperature of 24 °C. The time required for disintegration was recorded. According to the European Pharmacopoeia, oral lyophilizates pass the test if they disintegrate within 3 min.

### 2.3. QbD Approach

#### 2.3.1. Knowledge Space Development

The suitability of the proposed API for incorporation into OLs has been studied. Its physicochemical properties that could potentially affect the quality, safety, and efficacy of the drug product were studied. The scientific literature related to OLs, their properties, use, methods for their evaluation, and manufacture process was thoroughly studied. OLs that are currently available on the market were found using the search criteria for pharmaceutical forms. Their composition was carefully examined. The properties, purpose, role, and impact of incorporating the most commonly used excipients were studied. The impact of incorporating different proportions of these excipients into the formulation and the potential incorporation of other excipients that would help to improve the final properties of OLs were also examined. Findings of our experimental studies were studied and analyzed. The collected data were appropriately structured and visually presented.

#### 2.3.2. Definition of QTPP and Determination of the CQAs

Quality risk management tools were used to determine QTPP and CQA. The first step of the risk assessment process was the QTPP definition. When defining QTPP, we considered the utility and suitability of the product for the patient and the properties that are important for the therapeutic action or for clinical use. Considering the QTPP, the selection of CQAs followed. Their choice was further justified. The results of the risk assessment enabled identification of critical factors—CMA and CPP, which affect CQA. The aim of CMA and CPP identification is to prevent the implementation of unnecessary and inefficient phases in the development process [3,11,14,15].

#### 2.3.3. Risk Identification Process

By using risk identification, the information to identify hazards that relate to a risk question or description of a problem was systematically used. In the risk identification process, a risk question, i.e., “What can go wrong?”, was used. Risks were identified based on previously acquired knowledge by establishing the knowledge space. To identify potential factors that might adversely affect the quality of the drug product or its manufacturing process, the Ishikawa diagram was formed. Factors that could possibly adversely affect the selected parameters were chosen considering the previously gained knowledge [16,17].

#### 2.3.4. Risk Analysis and Assessment

**Figure 1:**
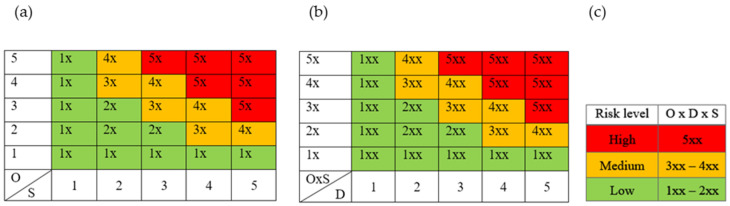
Risk analysis and risk assessment: (**a**) color matrix 1 with risk level descriptors O and S; (**b**) color matrix 2 with risk level descriptors O × S and D; (**c**) risk levels (O—occurrence probability; S—severity; D—detectability of errors). Detailed definition of risk levels is given in Table 2.

Risks were analyzed using the FMEA. The results of the final risk assessment were presented quantitatively by calculating the risk priority number (RPN) for each parameter that could potentially affect the CQAs. RPN was calculated by multiplying the values for occurrence probability (O), severity (S), and detectability (D) of errors (Table 2) and consequences [14]. A table in which the individual processes, potential risks, factors exposed to risks, and potential causes of errors are listed was created. O, S, and D values were determined using empirical findings and experiences described in various scientific articles. The values of O, S, and D were estimated, and the choice of individual values was duly justified [18]. In the first step of the qualitative risk assessment, color matrix 1 was developed (Figure 1a), which considers the values of O and S for each risk factor. This gave an intermediate value (e.g., Xx), which was considered in the next step. In the second step, color matrix 2 was created (Figure 1b), which considers not only the intermediate value (O × S) but also the value D of each risk factor. This gave the final value of the risk factor (e.g., Xxx). Considering the final value, a qualitative risk descriptor (low, medium, or high) was determined using Figure 1c [14,19,20]. High-risk factors were chosen as potential CPPs and CMAs.

#### 2.3.5. Design Space Establishment

Design space was established using the Minitab^®^ (Minitab Inc., State College, PA, USA) 17 software based on the previous experimental study, which comprised several testing formulations. Due to the compositions of the OLs present on the market, gelatin as a binder and mannitol as a filler were chosen for the lead formulation. Also, the effectiveness of PVP K25 as a substitute for gelatin was examined. The data gained were analyzed and evaluated using the response surface method and factorial design (2 × 4). As factors, concentration of excipients (% E, *w*/*w*) and concentration (*w*/*w*) of gelatin, mannitol, and PVP K25 were selected, while as a response, disintegration time of OLs was selected. The minimum and maximum values of the factors were determined. Using Minitab^®^, a linear regression model was designed, and the R^2^, R^2^ (adj), and R^2^ (pre) values were deduced. A factorial diagram that displays direction and strength of the influence of the formulation composition on disintegration time of OLs was formed and visually presented by response surfaces. Minitab^®^, with data input from experimental work, was used to further optimize the composition of OLs to achieve the shortest disintegration time. Value of desirability, which represents the matching of a response with its ideal value, was calculated. Suggestions obtained by Minitab^®^ were compared with the additional findings of our experimental work.

## 3. Results and Discussion

### 3.1. Knowledge Space

#### 3.1.1. Active Pharmaceutical Ingredient

Considering the first level of the anatomical therapeutic chemical (ATC) classification system, the largest share of OLs available on the market contains APIs with effects on the nervous system (N) (36%). These are followed by OLs classified as medicinal products for gastrointestinal and metabolic diseases (A) (23%) and miscellaneous medicinal products (V) (14%). There are at least 9% of OLs on the market that contain APIs that are classified as drugs for diseases of the musculoskeletal system (M), those that are classified as hormonal drugs for systemic action—except for sex hormones and insulins (H), and drugs for respiratory diseases (R).

Olanzapine is a very poorly soluble in water and saliva, yellowish crystalline drug that belongs to Biopharmaceutical Classification System (BCS) class II. Olanzapine is an antipsychotic characterized by first-pass metabolism, with as much as 40% of the dose being metabolized before reaching the systemic circulation [2,3,14]. Various pharmaceutical forms on the market contain olanzapine, such as film-coated tablets, capsules, intramuscular injections, orodispersible tablets, and oral lyophilizates (OLs) [21].

#### 3.1.2. Excipients

**Figure 2:**
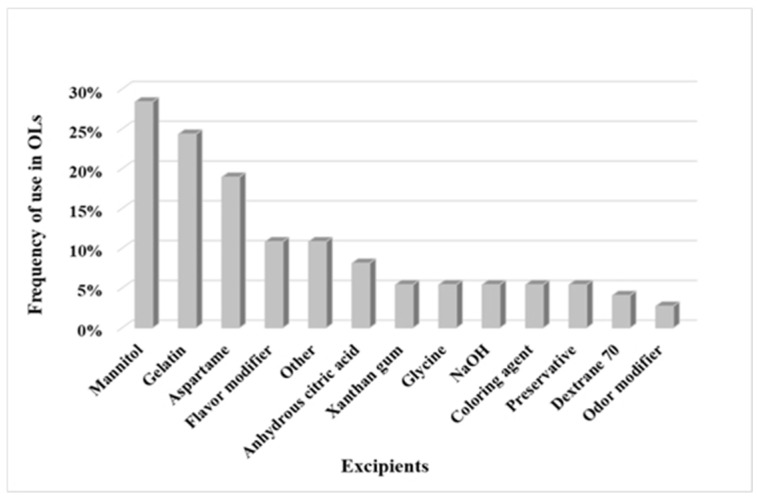
Frequency of individual excipients incorporated into OLs on the market. “Other”: excipients, which appeared only once.

A review of the composition of OLs on the market showed that the excipients that are most commonly found in formulations are mannitol (95%), as a matrix-forming agent/filler, and gelatin (69%) as a binder (Figure 2). Mannitol is a sugar alcohol in the form of a white, crystalline, odorless powder. One of the key advantages of mannitol is that it exhibits a high melting temperature of the mannitol/ice eutectic mixture (−1.5 °C), which enables an efficient lyophilization process and the production of physically stable OLs [22]. Gelatin is a biocompatible and biodegradable mixture of peptides and proteins. The incorporation of gelatin into the formulation allows lyophilization under more aggressive primary drying conditions (higher drying temperature), as it helps to increase the collapse temperature (Tc) of formulations [23,24]. Gelatin was found to prevent the fragility of OLs, but on the contrary, it can negatively affect their disintegration time [25,26].

#### 3.1.3. Manufacturing Process

**Figure 3:**
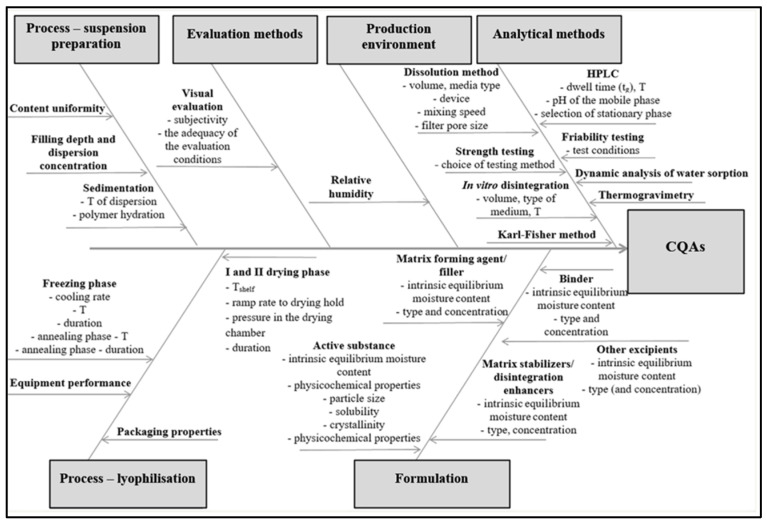
The Ishikawa diagram showing CQAs for OLs.

The Ishikawa diagram (Figure 3) shows the critical process parameters that can affect the manufacturing process and thus the final properties of OLs [12,14,17,24,27]. It was found that the most important parameter in the freezing phase is the cooling rate. Slower cooling of the dispersion (e.g., 0.5 °C/min) usually allows the formation of larger and more homogeneous ice crystals. It also affects the crystallization of excipients and enables faster and more efficient primary and secondary drying. To ensure the transition of the dispersion from liquid to solid state, the shelf temperature during freezing must be lower than the glass transition temperature of maximally freeze-concentrated solutions (Tg′) or the eutectic temperature (Te). It is important that the sample is completely frozen, as this achieves immobilization in terms of its structure, size, and shape and also slows down the degradation reactions [28]. Primary drying, as the most time- and energy-intensive stage, is a function of chamber pressure and shelf temperature, which directly affect the product temperature (Tp). To optimize the primary drying phase, the formulation as well as process parameters have to be considered. The most critical formulation parameter is Tc, which represents the highest acceptable Tp during primary drying, ensures the appropriate appearance of lyophilizates, and is closely related to Tg′. By including excipients that increase Tg′ and Tc, primary drying can be performed at higher temperatures, thus reducing the duration of this step significantly. Tp may also be higher than Tg′ (aggressive primary drying), provided that the drying process and formulation composition are optimized accordingly. The thermal conductivity of materials and the contact surface between containers and thermoregulated shelves are also important, as this affects the heat transfer to the containers and further to the product [24,29,30]. Important parameters of the secondary drying phase are shelf temperature and drying time. It is generally accepted to carry out secondary drying for a shorter time at a higher shelf temperature than vice versa. The rate of desorption decreases rapidly with time, so drying for longer than 6 h has no significant effect on reducing the water content in the sample. After the secondary drying is completed, the water content in the sample is 1–2% (*w*/*w*) [24,29,30].

### 3.2. Risk Identification

In terms of risk identification, QTPP was defined first, followed by the selection of CQAs. Table 3 presents selected QTPPs and their justifications.

In Table 4, product quality attributes were defined and CQAs selected. The appearance of OLs was chosen as a CQA since any change in appearance may indicate that there has been a change in quality. A change in appearance may be accompanied by changes in moisture content, disintegration time, or mechanical strength [31]. From the patient’s perspective, they may have concerns about the quality of therapy they are receiving. Mechanical strength was determined as one of the CQAs, as poor mechanical resistance is usually one of the major disadvantages of OLs, which limits their handling [6,7]. Dissolution was evaluated as the next CQA because the aim was to develop OLs that allow faster and more efficient dissolution of API and thereby improve its bioavailability. The rate of absorption and/or the extent of bioavailability of olanzapine are controlled by the rate at which it dissolves in gastrointestinal fluids. The oral bioavailability of olanzapine is very poor, which is associated with poor water/saliva solubility and an insufficient dissolution rate. Olanzapine solubility and dissolution rate are enhanced due to its amorphous form in porous OLs [2,3]. Moisture residue was chosen as a CQA as it can significantly affect the stability of OLs and the stability of API itself [32]. Disintegration time was chosen as a CQA, as it is one of the criteria of the European Pharmacopoeia for the acceptability of manufactured OLs. OLs must disintegrate in less than three minutes. Extremely fast disintegration leads to faster dissolving of a drug substance, which enables improved absorption and bioavailability as well as a rapid onset of therapeutic effect. Rapid disintegration also allows easier and faster drug administration and, thus, greater patient compliance. A literature review revealed that it is difficult to develop a formulation that allows for the appropriate mechanical strength of OLs and also their fast disintegration [1,6,7]. Using the knowledge gained, an Ishikawa diagram (Figure 3) showing material attributes and process parameters that can affect CQAs of OLs was made.

### 3.3. Risk Analysis and Assessment

Risks were analyzed and assessed using failure mode and effect analysis (FMEA). The reliability of instruments and analytical methods for the evaluation of OLs was not included in the analysis as it was identified as a negligible risk factor, considering that the instruments are periodically calibrated and qualified and the analytical methods are periodically validated [33]. The calculated RPN values are shown in Table 5. The higher the RPN value, the greater the risk posed by a single factor, and vice versa. However, the values of O, S, D, and RPN can change considerably with time and the progress of knowledge. In general, the more knowledge gained, the lower the risk. The level of risk can also be reduced with an effective CPP and CMA control strategy, as well as through continuous product and process improvements [14,15,19]. FMEA results need to be critically interpreted, since if a low value of RPN is calculated while the severity of consequences is high, a falsely reduced level of risk can occur. Therefore, if the same RPN values are calculated for two individual consequences of two different risk factors, the higher the S value, the higher the risk level. Additionally, qualitative risk assessment was performed (Table 5), which also enabled better transparency of the risk assessment outcomes [14,15,19]. Spaces with potential CMAs and CPPs were colored dark gray. As potential CMAs, unsuitable concentration and/or substance and/or intrinsic equilibrium moisture content of matrix forming agent/filler, binder, or matrix stabilizer, together with particle size, crystallinity/amorphousness, and solubility of API, were determined. As CPPs, cooling rate, pressure in the drying chamber, duration, and shelf temperature of primary and secondary drying were specified. If not properly controlled, all listed parameters can adversely affect disintegration time, mechanical resistance, and the appearance of OLs.

### 3.4. Design Space Establishment

A preliminary risk assessment is usually followed by DoE, which is a structured and organized method for determining the exact relationship between factors that influence a product and its manufacturing process. In this research, the basic principles of DoE were applied. Design space was established using the Minitab^®^ 17 software using the linear regression method and a 2-level 4-factor experimental design.

#### R^2^, R^2^ (pre), R^2^ (adj)

**Figure 4:**
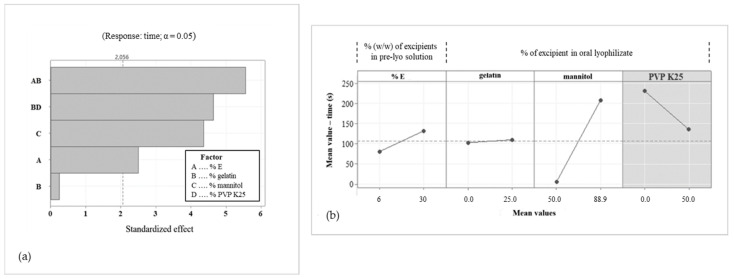
Pareto chart showing influence of formulation composition on redispersion time (**a**) and factorial graph showing influence of factors on disintegration time (Minitab^®^) (**b**).

**Figure 5:**
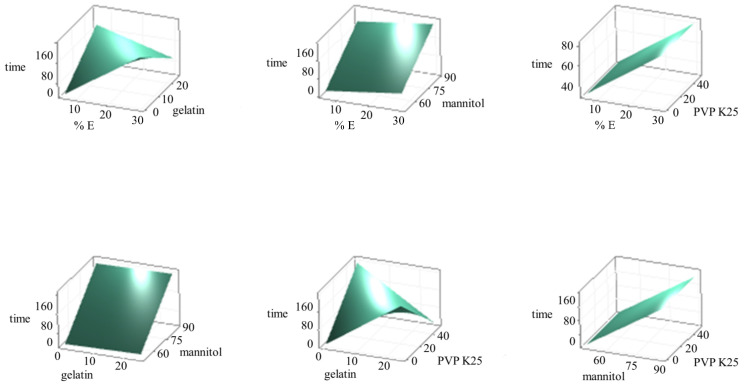
Surface diagrams showing influence of factors and their combinations on the disintegration time of OLs (Minitab^®^). % E—The total concentration of excipients in the pre-lyophilized liquid formulations; gelatin, mannitol, PVP K25—% in OL.

In the initial phase of designing a model, all possible interactions between the total amount of excipients (% of E), gelatin concentration, mannitol concentration, and PVP K25 concentration, as well as each individual listed parameter, were considered factors. Since a model with low predictive power was obtained, only two-factor interactions with a significant effect (*p* < 0.05) on the response (disintegration time) were further studied. It is known that the poor predictive power of the model is usually a consequence of the excessive number of factors included in the model [34]. Once a linear regression model is created, its value is assigned through the coefficients given by the program. One of the commonly used coefficients is the coefficient of determination (R^2^), which measures the percentage of response variations that can be explained by a set model. In fact, R^2^ describes how well a model fits our data. The higher the R^2^, the more response variations can be explained by the model. R^2^ is between 0% and 100%. The drawback of using R^2^ is that its value can increase if more factors are included in the model, making the model misleadingly more satisfactory. This can be addressed by utilizing adjusted R^2^-R^2^ (adj) and predicted R^2^-R^2^ (pre). R^2^ (adj) is the percentage of the variation in the response that is explained by the model, adjusted for the number of predictors in the model relative to the number of observations, while R^2^ (pre) is used to determine how well the set model predicts the response for new observations. Regarding our model, the obtained value of R^2^ was 88.69%, which indicates that the model can explain 88.69% of all response variations. The R^2^ (adj) was 86.52% and thus comparable with R^2^, and, furthermore, the R^2^ (adj) did not decrease significantly with the inclusion of individual factors, which explains that the factors used in the model are relevant to it. If R^2^ (adj) decreases with respect to R^2^ and includes individual factors in the model, this means that the latter can be excluded from the model. The value of R^2^ (adj) means that 13.48% of all variations cannot be explained by the set model. The R^2^ (pre) was 81.19%, which represents the degree of model prediction of the redispersion time in the case of altered factors, such as the total amount of excipients, concentration of gelatin, mannitol, or PVP K25 in OLs [13]. The influence of factors on the response is more clearly explained by Figure 4 and Figure 5. The Pareto chart in Figure 4a, which was designed based on the experimental results and considering the risk aspects, shows the influence of formulation composition (factors) on disintegration time (response).

The program Minitab^®^ was not able to assess the impact of PVP K25 alone; therefore, it was not included in the Pareto chart. Obtained results revealed that interaction “% E*gelatin” has the greatest influence on the disintegration time of OLs, followed by interaction “gelatin*PVP K25”, effect of “mannitol”, effect of “% E” and far below the standardized effect, the minor effect of “gelatin”. The factorial graph (Figure 4b) shows that a higher total amount of excipients as well as an increase in mannitol concentration in the formulation significantly contribute to the prolongation of the disintegration time, while the gelatin impact is not strongly pronounced. During the laboratory experimental part, an opposite trend was observed for PVP K25, but as its effect was not recognized as significant (*p* > 0.05), PVP K25 was not included in the model. Nevertheless, it was found out that due to the excessive fragility of OLs with only PVP K25, gelatin has to be added. Gelatin is also very important for the incorporation of olanzapine, which is a very poorly water-soluble drug. By constructing surface diagrams, it is possible to evaluate the simultaneous influence of two factors while the others remain constant. As a factorial graph, surface diagrams on Figure 5 clearly show that increasing the total amount of excipients prolongs the OL’s disintegration time, which was in line with our expectations. The interactions “% E*mannitol”, “gelatin*mannitol”, and “mannitol*PVP K25” did not have a significant effect on the disintegration time and did not prevail over the influence of mannitol itself (Figure 5). In the case of the “% E*PVP K25” interactions, it can be concluded that the addition of PVP K25 to the formulation allows a shorter disintegration time to be achieved compared with a formulation without PVP K25, while additional variations in the amount of PVP K25 did not alter the disintegration time of OLs. An interesting effect was observed for interactions “% E*gelatin” and “gelatin*PVP K25”.

Namely, at a sufficiently low amount of excipients (up to 20%), when the concentration of mannitol is also relatively low, the addition of gelatin (up to 15%) leads to an increase in the disintegration time, but still within the prescribed limit. With a simultaneous increase in the total amount of excipients and gelatin, the disintegration time of OL increases, but when the concentration of mannitol is sufficiently reduced, disintegration time interestingly starts to decrease at the expense of the increase in the concentrations of gelatin. Further, if gelatin concentration is low enough, addition of PVP K25 prolongs the time for disintegration of OLs, while at the point when concentration of gelatin and PVP K25 combined prevail over mannitol concentrations, disintegration time drops. In brief, mannitol concentration is crucially important regarding the disintegration of OLs.

### 3.5. OLs Formulation Optimization

The aim of this section was to define an optimized OL formulation, utilize software as well as an empirical laboratory approach, and compare the obtained responses. Considering the previously designed model, the algorithm behind Minitab^®^ proposed five different OL compositions relating to concentrations of gelatin, mannitol, and PVP K25 (Table 6). Among them, four compositions were assessed with a desirability value of 1, which means that they enable obtaining OLs with an ideal response, i.e., disintegration time. All formulation compositions proposed by Minitab^®^ contain 6% (*w*/*w*) excipients—mannitol and PVP K25—without gelatin, among which mannitol predominates. Regardless of the ratio between mannitol and PVP K25 in the OL’s composition, the software predicts equal values of responses (9 s). As mentioned, the model proposals were compared with laboratory findings to identify potential discrepancies between them. Laboratory experiments revealed that the lower the content of excipients, the faster the OL disintegrates but exhibits a higher degree of fragility. Here, the main shortcoming of the set model was noted, namely that the appearance of OLs as a response was not incorporated in the model. Regardless of the type of excipients, all lyophilizates began to show reduced disintegration ability at an excipient concentration above 15% (*w*/*w*). Lyophilizates, which contained PVP K25 in addition to mannitol, showed appropriate disintegration times but had drawbacks such as cracking and increased fragility. The designed model gave comparable composition directions (Table 6), but considering only disintegration time, it also proposed compositions with an insufficient amount of mannitol (below 70%, *w*/*w*), together with no gelatin, which in practice led to the collapse of OLs. The shortcoming of the model was reflected in the fact that, due to the probability of gelatin prolonging the time required for OL disintegration recognized by the model, the proposed OL compositions did not include gelatin. Therefore, we stated that the set model needs to be further optimized. The disadvantages of formulations with PVP K25 and mannitol were solved by the addition of gelatin, which strengthened the framework of the OLs and thus prevented their crumbling and cracking without significant disintegration time extensions. Gelatin is important from the viewpoint of providing the visual appearance of OLs and handling them, as well as enabling the dispersion of poorly water-soluble drugs, which was found out during further laboratory work. However, experimentally obtained results, which consider the appearance and disintegration time of the OLs, revealed the most suitable compositions, presented in Table 6. Once the most optimal compositions of OLs were determined, the incorporation of a drug, i.e., olanzapine, followed. The inclusion of olanzapine slightly shortened the disintegration time of OLs with a gelatin:mannitol:PVP K25 mass ratio of 0.5/1:5:2, compared with OLs without API. We hypothesized that since olanzapine is not soluble in water, it reduces the number of bonds between the water-soluble excipient particles, which contributes to the disintegration of the lyophilizate. Regardless of the presence of olanzapine, all OLs with a gelatin concentration higher than 15% (*w*/*w*) had disintegration times above 180 s. A comprehensive comparison of the results showed that the optimal composition of the OLs is a balance between their disintegration time and appearance. Accordingly, we concluded that the most appropriate OLs are prepared from pre-lyophilized liquid formulations containing 6% (*w*/*w*) of excipients, which are in a mass ratio of gelatin: PVP K25: mannitol of 1:2:5, whether with or without drug (Table 6).

## 4. Conclusions

To date, analysis and risk assessments, along with FMEA, of pharmaceutical products manufactured by lyophilization are not widespread in the scientific literature. The present article aimed to explore the potential of incorporating a QbD approach at the laboratory level, specifically to establish a platform for the preparation of drug-containing oral lyophilizates. The study revealed the most important CQAs, such as the appearance and disintegration time of oral lyophilizates, which directly depend on CMAs and CPPs. Among others, type and concentration of excipients and solubility of the active substance were defined as CMAs, while CPPs are combinations of cooling rate, shelf temperature, and chamber pressure during drying. In accordance with the laboratory results, the set model also confirmed that the optimal concentration of excipients in the pre-lyophilized liquid formulation is 6% (*w*/*w*) and that the predominant component should be mannitol. It was demonstrated that the optimal ratio of mannitol:PVP K25 is 5:2, further confirmed by Minitab^®^ as well. It was concluded that the set model represents an approximation of the values of responses, while in the case of implementation in further work, it should be somewhat optimized. The contribution of our study to the existing scientific knowledge in the field of OLs, specifically the selection of suitable excipients according to API properties, represents the basis for establishing a QbD approach for the potential production of OLs with olanzapine. The added value of the study is the finding that the composition of OLs with olanzapine is an interplay between the amount of gelatin, appearance, and disintegration time of OLs. We assume that certain findings will fill the knowledge gap related to the solubility problems of new APIs.

## Figures and Tables

**Table 1:** Tested formulations of OLs with disintegration times and appearance acceptability.

% E [*w*/*w*]	% of Gelatin in Lyophilizate	% of Mannitol in Lyophilizate	% of PVP K25 in Lyophilizate	Disintegration Time [s]	Appearance Acceptability
30	11.1	88.9	0	>180	NO
30	16.7	83.3	0	>180	NO
30	25	75	0	>180	NO
25	16.7	83.3	0	>180	NO
20	16.7	83.3	0	>180	NO
15	16.7	83.3	0	>180	NO
10	16.7	83.3	0	>180	NO
6	16.7	83.3	0	>180	NO
30	0	50	50	>180	NO
30	0	83.3	16.7	>180	NO
25	0	83.3	16.7	>180	NO
20	0	50	50	>180	NO
15	0	50	50	39	NO
15	0	83.3	16.7	31	NO
15	0	88.9	11.1	24	NO
15	0	71.4	28.6	26	NO
15	0	62.5	37.5	16	NO
10	0	50	50	15	NO
10	0	83.3	16.7	17	NO
10	0	88.9	11.1	17	NO
10	0	71.4	28.6	21	NO
10	0	62.5	37.5	11	NO
6	0	50	50	9	NO
6	0	83.3	16.7	15	YES
6	0	88.9	11.1	15	YES
6	0	71.4	28.6	15	YES
6	0	62.5	37.5	10	NO
6	7.7	76.9	15.4	12	YES
6	6.7	80	13.3	9	YES
6	14.3	71.4	14.3	22	YES
6	12.5	62.5	25	25	YES

% E—The total concentration of excipients in the pre-lyophilized liquid formulations.

**Table 2:** Occurrence probability, severity level, detectability determination, and their justification.

(a) Occurrence Probability (O)	Level	Justification
**5**	Very likely	An error was detected in several important experiments
**4**	Probably	An error was detected in some important experiments
**3**	Unlikely	An error was detected in some important experiments, but the probability of its occurrence is minimal if the procedures are performed according to previously known instructions
**2**	Almost impossible	An error occurred in one or two important experiments
**1**	Practically impossible	The error has never occurred; its occurrence is theoretically possible
**(b) Severity** **(S)**		
**5**	Catastrophic	The error has a great impact on the quality of the product; reprocessing is not possible
**4**	Critical	The error has a great impact on the quality of the product; reprocessing is possible
**3**	Serious	The error has an impact on the quality of the product, but it can be corrected by recycling
**2**	Not serious	The error has no effect on the quality of the product
**1**	Negligible	The error has no effect on product quality or process robustness
**(c) Detectability (D)**		
**5**	Difficult to detect	There is no possibility of detecting an error
**4**	Low probability of detection	Non-validated automatic or manually operated fault detection system (e.g., visual inspection) exists
**3**	Moderate probability of detection	A validated manually controlled system for indirect fault detection exists (e.g., process analysis technology (PAT) measurements or in-process control of parameters not directly related to the fault)
**2**	Easier to detect	A validated, manually controlled system for direct error measurement exists
**1**	Easy	1. A validated automatic system for direct error measurement exists 2. There are two or more validated manually operated sensing systems; direct or indirect detection (e.g., control area or in-process control)

**Table 3:** Selected QTPP and their justification.

QTPP		Target	Justification
**Route of administration**		Oral	Ease of taking medications, patient compliance
**Pharmaceutical form**		Oral lyophilizate	Disintegration time <3 min, ease of administration, no water required, prevention of patient suffocation
**Strength (olanzapine)**		5 mg	Desired dose in pharmaceutical form, safety, efficacy
**Quality attributes**	Physical properties	No visible defects, uniformity of dosage units, no visible collapse of the structure	Aesthetic appearance and desired functionality
	In vitro dissolution	e.g., at least 90% in 180 s	Rate of onset of therapeutic action, bioavailability
	Disintegration time	<3 min	Intact pharmaceutical form, consumption prevention, suffocation of the patient prevention, non-consumption prevention, ease of taking, dissolving active substance
	Mechanical resistance	≥15 N	Packaging selection, handling, storage conditions
	Wetting time	≤15 s	Redispersion rate, dissolution time
	Content	95–105%	Safety, efficacy, quality
**Primary packaging**		Suitable for storage under normal conditions	Ensures product integrity throughout storage time

**Table 4:** Definition and selection of CQAs.

Product Quality Attributes		Target	Is This CQA?
**Physical properties**	Appearance	No visible defects, uniformity of dosage units, no visible collapse of the structure	**YES**
	Odor	No unpleasant odor	NO
	Size	Suitable	NO
	Mechanical resistance	≥15 N	**YES**
**In vitro dissolution**		e.g., at least 90% in 180 s	**YES**
**Disintegration time**		<3 min	**YES**
**Wetting time**		≤15 s	NO
**Moisture residue**		<1%	**YES**

**Table 5:** Risk analysis and assessment using FMEA. Potential CMAs and CPPs are colored in dark gray.

		Potential Risk	“What Can Go Wrong?”	RPN Calculation			
				O	S	D	RPN
**Formulation Parameters**	Drug Binder Filler Disintegrant Other excipients	Quality, safety, efficiency of the drug	Unsuitable concentration and/or substance, intrinsic equilibrium moisture content	**4**	**5**	**3**	**60**
				**5**	**5**	**3**	**75**
				**5**	**5**	**3**	**75**
				**5**	**5**	**3**	**75**
				**2**	**3**	**2**	**12**
		Inadequate mechanical resistance, instability of drug, influence on Bioavailability	Particle size	**4**	**5**	**3**	**60**
			Crystallinity/amorphousness	**4**	**5**	**3**	**60**
			Solubility	**4**	**5**	**3**	**60**
			Intrinsic equilibrium moisture content	**2**	**4**	**2**	**16**
			Incomplete hydration of the polymer	**3**	**4**	**3**	**36**
			Temperature	**2**	**4**	**3**	**24**
**Process Parameters**	Freezing phase	Formation of small and inhomogeneous ice crystals	Cooling rate	**4**	**4**	**4**	**64**
		Quality, safety, efficiency of the drug	Temperature	**3**	**4**	**3**	**36**
			Duration	**2**	**4**	**3**	**24**
			Annealing phase—temperature	**3**	**3**	**2**	**18**
			Annealing phase—duration	**3**	**3**	**2**	**18**
	Primary drying	Tp > Tc, loss of porous structure	Ramp rate to drying hold	**2**	**2**	**3**	**12**
			Shelf temperature	**4**	**4**	**3**	**48**
			Pressure in the drying chamber	**4**	**4**	**3**	**48**
			Filling depth	**2**	**4**	**3**	**24**
			Highly concentrated samples	**3**	**4**	**2**	**24**
			Duration	**4**	**4**	**3**	**48**
	Secondary drying	Tp > Tc, loss of porous structure, high moisture residue	Shelf temperature	**4**	**4**	**3**	**48**
			Pressure in the drying chamber	**2**	**2**	**2**	**8**
			Duration	**4**	**4**	**4**	**64**
			Ramp rate to drying hold	**3**	**4**	**3**	**36**

O—occurrence probability; S—severity; D—detectability of errors; RPN—risk priority number; Tp—product temperature; Tc—collapse temperature.

**Table 6:** Oral lyophilizate compositions (in %) proposed by Minitab^®^ and experimentally assessed. The total concentration of excipients in the pre-lyophilized liquid formulation was 6% (*w*/*w*).

%	Gelatin	Mannitol	PVP K25	API [mg/mL]	Disintegration Time [s]
MINITAB^®^	0.0	72.60	27.39	/	9
	0.0	88.90	11.10	/	9
	0.0	68.98	31.02	/	9
	0.0	51.79	48.21	/	9
	0.0	87.33	12.67	/	9
Experimentally Obtained	0.0	83.3	16.7	/	15
	0.0	88.9	11.1	/	15
	0.0	71.4	28.6	/	15
	7.7	76.9	15.4	/	12
	6.7	80.0	13.3	/	9
	14.3	71.4	14.3	/	22
	**12.5**	**62.5**	**25**	**/**	**25**
	6.7	80.0	13.3	2.5	5
	**12.5**	**62.5**	**25**	**2.5**	**5**

The most appropriate OL composition with and without API is printed in bold.

## Data Availability

Data will be made available on request.

## References

[1] (2019). European Pharmacopoeia.

[2] Dixit M., Kini A.G., Kulkarni P.K. (2011). Enhancing the aqueous solubility and dissolution of olanzapine using freeze-drying. Brazilian J. Pharm. Sci..

[3] Elly Lilli Canada Inc (2020). Zyprexa. http://pi.lilly.com/ca/zyprexa-ca-pm.pdf.

[4] Ghosh T., Ghosh A., Prasad D. (2011). A review on new generation orodispersible tablets and Its future prospective. Int. J. Pharm. Pharm. Sci..

[5] Nagar P., Singh K., Chauhan I., Verma M., Yasir M., Khan A., Sharma R., Gupta N. (2011). Orally disintegrating tablets: Formulation, preparation techniques and evaluation. J. Appl. Pharm. Sci..

[6] PharmTech. http://www.pharmtech.com/orally-disintegrating-tablets-effect-recent-fda-guidance-odt-technologies-and-applications.

[7] Slavkova M., Breitkreutz J. (2015). Orodispersible drug formulations for children and elderly. Eur. J. Pharm. Sci..

[8] Al Husban F.A., El Shaer A.M., Jones R.J., Mohammed A.R. (2010). Recent patents and trends in orally disintegrating tablets. Recent Pat. Drug Deliv. Formul..

[9] Patil P.B., More V.N., Tour N.S. (2015). Recent Trends in Orodispersible Tablets—An Overview of Formulation Technology and Future Prospects. Int. J. Pharma Sci. Res..

[10] Lyophilization—The Key to Creating Acceptable & Effective Fast-Dissolving Oral Formulations. https://drug-dev.com/lyophilization-the-key-to-creating-acceptable-effective-fast-dissolving-oral-formulations/.

[11] EMEA/CHMP ICH Topic Q8 (R2) Pharmaceutical Development, Step 5: Note for Guidance on Pharmaceutical Development. http://www.ema.europa.eu/docs/en_GB/document_library/Scientific_guideline/2009/09/WC500002872.pdf.

[12] Porfire A., Tomuta I., Iurian S., Casian T. (2019). Quality by Design Considerations for the Development of Lyophilized Products. Pharmaceutical Quality by Design.

[13] Preskar M., Videc D., Vrečer F., Gašperlin M. (2021). Investigation of design space for freeze-drying injectable ibuprofen using response surface methodology. Acta Pharm..

[14] Radhakrishnan V., Davis P., Hiebert D., Warne N., Mahler H.C. (2018). Scientific approaches for the application of QbD principles in lyophilization process development. Challenges in Protein Product Development.

[15] Manteghi R., Pallagi E., Olajos G., Csóka I. (2020). Pegylation and formulation strategy of Anti-Microbial Peptide (AMP) according to the quality by design approach. Eur. J. Pharm. Sci..

[16] European Medicines Agency (EMA) (2014). ICH Guideline Q9 on Quality Risk Management.

[17] Suciu Ș., Iurian S., Bogdan C., Iovanov R., Rus L., Moldovan M., Tomuţă I. (2018). QbD approach in the development of oral lyophilisates with ibuprofen for paediatric use. Farmacia.

[18] Casian T., Iurian S., Bogdan C., Rus L., Moldovan M., Tomută I. (2017). QbD for pediatric oral lyophilisates development: Risk assessment followed by screening and optimization. Drug Dev. Ind. Pharm..

[19] Kotak M. Pharma Project Risk Management. https://www.slideshare.net/kotakmegha/pharma-project-risk-management.

[20] Pallagi E., Ismail R., Paál T.L., Csóka I. (2018). Initial Risk Assessment as part of the Quality by Design in peptide drug containing formulation development. Eur. J. Pharm. Sci..

[21] Lai F., Pini E., Corrias F., Perricci J., Manconi M., Fadda A.M., Sinico C. (2014). Formulation strategy and evaluation of nanocrystal piroxicam orally disintegrating tablets manufacturing by freeze-drying. Int. J. Pharm..

[22] Anko M., Bjelošević M., Planinšek O., Trstenjak U., Logar M., Ahlin Grabnar P., Brus B. (2019). The formation and effect of mannitol hemihydrate on the stability of monoclonal antibody in the lyophilized state. Int. J. Pharm..

[23] Baheti A., Kumar L., Bansal A.K. (2010). Excipients used in lyophilization of small molecules. J. Excipients Food Chem..

[24] Bjelošević M., Zvonar Pobirk A., Planinšek O., Ahlin Grabnar P. (2020). Excipients in freeze-dried biopharmaceuticals: Contributions toward formulation stability and lyophilisation cycle optimisation. Int. J. Pharm..

[25] Jones R.J., Rajabi-Siahboomi A., Levina M., Perrie Y., Mohammed A.R. (2011). The influence of formulation and manufacturing process parameters on the characteristics of lyophilized orally disintegrating tablets. Pharmaceutics.

[26] Lin W., Yan L., Mu C., Li W., Zhang M., Zhu Q. (2002). Effect of pH on gelatin self-association investigated by laser light scattering and atomic force microscopy. Polym. Int..

[27] De Beer T.R.M., Wiggenhorn M., Hawe A., Kasper J.C., Almeida A., Quinten T., Friess W., Winter G., Vervaet C., Remon J.P. (2011). Optimization of a pharmaceutical freeze-dried product and its process using an experimental design approach and innovative process analyzers. Talanta.

[28] Patel Z.K., Patel R.R., Patel K.R., Patel M.R. (2014). A Review: Formulation of Fast Dissolving Tablet. PharmaTutor..

[29] Kasper J.C., Friess W. (2011). The freezing step in lyophilization: Physico-chemical fundamentals, freezing methods and consequences on process performance and quality attributes of biopharmaceuticals. Eur. J. Pharm. Biopharm..

[30] Bjelošević M., Seljak K.B., Trstenjak U., Logar M., Brus B., Ahlin Grabnar P. (2018). Aggressive conditions during primary drying as a contemporary approach to optimise freeze-drying cycles of biopharmaceuticals. Eur. J. Pharm. Sci..

[31] Patel S.M., Nail S.L., Pikal M.J., Geidobler R., Winter G., Hawe A., Davagnino J., Rambhatla Gupta S. (2017). Lyophilized Drug Product Cake Appearance: What Is Acceptable?. J. Pharm. Sci..

[32] ParmTech Residual Moisture Testing Methods for Lyophilized Drug Products. www.pharmtech.com/residual-moisture-testing-methods-lyophilized-drug-products.

[33] Sangshetti J.N., Deshpande M., Zaheer Z., Shinde D.B., Arote R. (2017). Quality by design approach: Regulatory need. Arab. J. Chem..

[34] How to Interpret Adjusted R-Squared and Predicted R-Squared in Regression Analysis. https://statisticsbyjim.com/regression/interpret-adjusted-r-squared-predicted-r-squared-regression.

